# A Fatal Case of Congenital Langerhans Cell Histiocytosis with Disseminated Cutaneous Lesions in a Premature Neonate

**DOI:** 10.1155/2016/4972180

**Published:** 2016-10-19

**Authors:** Michio Inoue, Yoko Tomita, Tsuyoshi Egawa, Tomoaki Ioroi, Masaaki Kugo, Shinsaku Imashuku

**Affiliations:** ^1^Department of Pediatrics, Japanese Red Cross Society Himeji Hospital, Himeji, Hyogo 670-8547, Japan; ^2^Department of Child Neurology, National Center Hospital, National Center of Neurology and Psychiatry, Tokyo 187-8551, Japan; ^3^Department of Laboratory Medicine, Uji-Tokushukai Medical Center, Uji, Kyoto 611-0042, Japan

## Abstract

*Background*. The outcome of neonates with congenital cutaneous Langerhans cell histiocytosis (LCH) is variable.* Observations*. We report a case of LCH in a female premature neonate born at 33-week gestation. She had disseminated cutaneous lesions, which consisted of hemorrhagic papules and vesicles, with sparse healthy skin areas, and the hands and feet were contracted with scarring and blackened. She was in respiratory failure although no apparent pulmonary or bone lesions on X-rays were noted. Skin biopsy confirmed a diagnosis of LCH due to observation of CD1a^+^ Langerhans cells, which lacked expression of E-cadherin and CD56. The patient died 57 hours after birth.* Conclusions*. Based on this case and the literature survey, the outcome of premature babies with congenital cutaneous LCH lesions is noted to be unfavorable, with the majority of such cases suffering from multisystem disease.

## 1. Introduction

Langerhans cell histiocytosis (LCH) is characterized by lesions that include CD1a^+^CD207^+^ dendritic cells, along with inflammatory cell infiltrates. Molecular analysis clarified that LCH arises from pathological activation of the mitogen-activated protein kinase pathway in myeloid precursors [1]. In particular, the* BRAF V600E* mutation in the gene encoding serine/threonine-protein kinase B-raf has been identified in ~50–60% of patients with LCH [2]. The clinical features of LCH range from localized, single-organ lesions to multifocal, multiorgan lesions. These lesions can either regress spontaneously or progress aggressively; thus, LCH can have a range of outcomes that vary in severity from benign to fatal [3]. Fetal and neonatal LCH are approximately equally divided into two groups: LCH limited to skin and LCH involving multiple organs [4]. Congenital LCH that is limited to cutaneous lesions is generally thought to be clinically benign, with a good prognosis [5], but rare cases have a poor outcome [6, 7]. Notably, congenital cutaneous LCH in preterm babies (born before 37-week gestation) is a severe, systemic disease that usually causes death* in utero* or after delivery [8–12]. In some premature neonates with LCH, lethal hydrops fetalis may develop [8, 9]. Here, we report a fatal case of congenital LCH in a prematurely born female neonate with disseminated cutaneous lesions resembling severe burns, but with no apparent pulmonary or bone lesions.

## 2. Case Report

**Figure 1:**
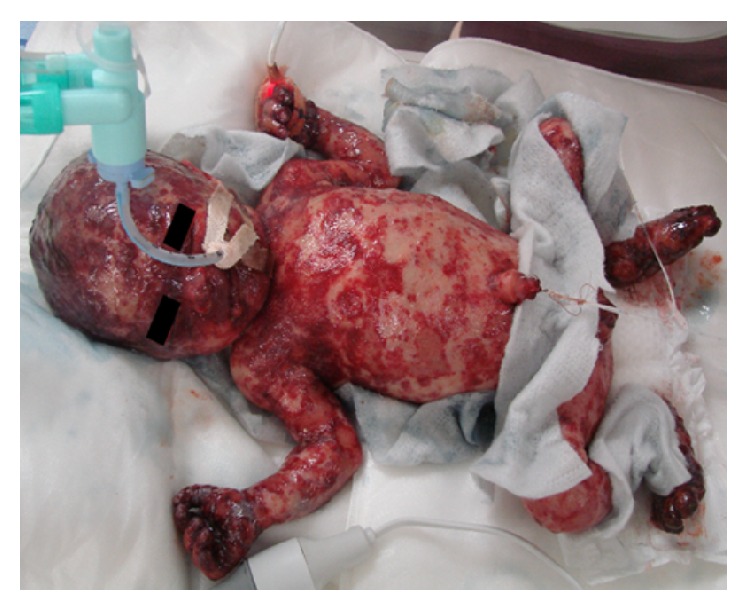
Photograph of the patient, showing extensive disseminated vesiculopapular cutaneous lesions. Hands and feet were contracted and blackened.

**Figure 2:**
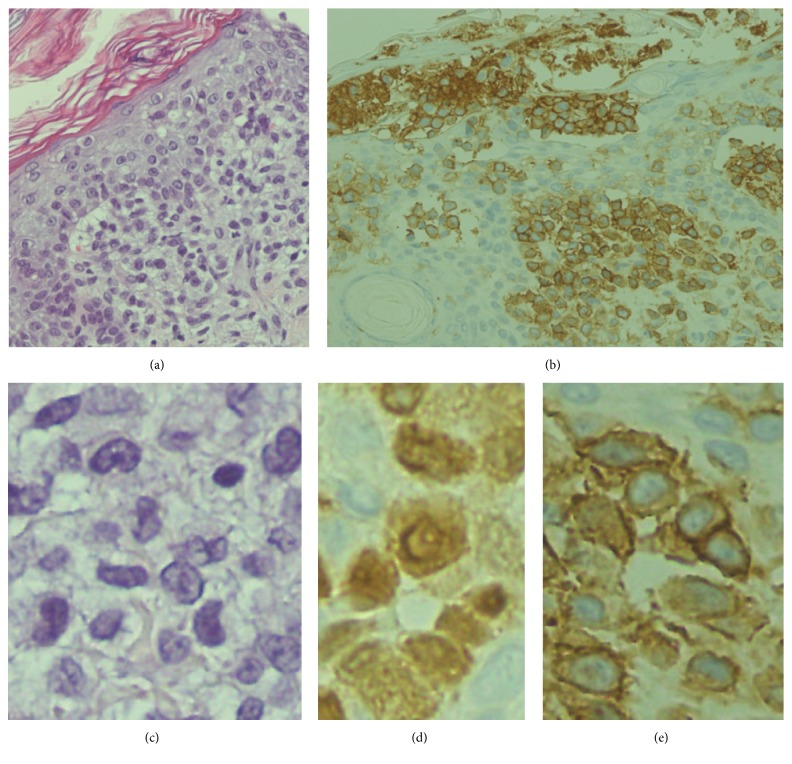
Histopathology of a cutaneous lesion showing histiocytic cell infiltration in the epidermis and dermis as well as under the stratum corneum ((a); HE stain), which were stained positive for CD1a (b) (original magnification, ×400), magnified photos showing characteristic folded “coffee bean” nucleus ((c); HE stain), positive S100 staining (d), and positive CD1a (e). Staining for E-cadherin and CD56 was negative (data not shown).

The mother of the patient was 33-year-old, gravida 2, and para 2, and the pregnancy was uneventful until 32-week gestation when she had a threatened premature labor and was admitted to a maternity hospital. She gave birth by vaginal delivery at 33-week and 3-day gestation. Fetal ultrasonography did not reveal any specific abnormalities, and myocardial contractility was assessed as normal by echocardiography. The mother, who was negative for toxoplasma, chlamydia, rubella, human papilloma virus, syphilis, and human T cell leukemia virus type 1, had been treated with flomoxef 1 week prior to delivery because of inflammatory signs (white blood cell count (WBC), 10,540/*μ*L, and C-reactive protein (CRP) level, 1.51 mg/dL). The newborn baby weighed 1,706 g and had Apgar scores of 8 at 1 min and 8 at 5 min. At birth, she was noted to have disseminated cutaneous lesions consisting of hemorrhagic papules and vesicles on the scalp, face, trunk, and extremities. Soon after birth, the baby was not anemic but showed hypoproteinemia (total protein, 3.4 g/dL; albumin, 2.2 g/dL) and respiratory failure. She was immediately intubated and transferred to our hospital. When the patient arrived the following were noted: temperature, 34.7°C; heart rate, 126 beats/min; SpO_2_, 100% (FiO_2_ 0.25); and blood pressure, 44/34 mmHg. Laboratory data revealed the following: WBC, 7,000/*μ*L; hemoglobin, 15.1 g/dL; platelet count, 97,000/*μ*L; total protein, 3.2 g/dL; albumin, 1.9 g/dL; aspartate aminotransferase, 30 U/L; alanine transaminase, 8 U/L; total bilirubin, 7.1 mg/dL; blood urea nitrogen, 11.1 mg/dL; creatinine, 0.61 mg/dL; sodium, 145 mEq/L; potassium, 4.3 mEq/L; calcium, 8.8 mg/dL; CRP, 0.76 mg/dL; IgG, 245 mg/dL; IgA, 0 mg/dL; IgM, 4 mg/dL; prothrombin time, 69%; PT-INR, 1.25; activated partial thromboplastin time, 72.0 s; fibrinogen, 238 mg/dL; fibrin degradation products, 23 *μ*g/mL; D-dimer, 10.0 *μ*g/mL; and antithrombin, 25%. On admission, the baby's skin had the appearance of severe burns, with widespread hemorrhagic papules and vesicles and sparse healthy skin. In particular, the hands and feet were contracted with scarring and blackened (Figure 1). Chest X-ray and bone scans revealed no abnormalities (data not shown).* Staphylococcus* species were cultured from plantar skin lesions, but no infectious agents were found in swabs taken from the oral mucosa. Blood cultures were not performed. The baby was given infusion fluid along with ampicillin, intravenous immunoglobulin, and albumin. Skin lesions were managed with procedures used for extensive burn care. Biopsy specimens from skin lesions were taken on Day 1 of admission. The results showed histiocytic cell infiltration in the epidermis and dermis as well as under the stratum corneum with proliferation of S100^+^, CD1a^+^ Langerhans cells, leading to a diagnosis of LCH (Figure 2). These cells did not express E-cadherin or CD56. Detection of the* BRAF V600E* mutation was attempted with DNA extracted from paraffin-embedded sections of skin biopsy specimens, but the result was negative (data not shown). The patient did not respond well to intensive therapeutic measures under respiratory care, including ceftriaxone, fluconazole, and catecholamine, as well as attempted correction of metabolic acidosis. On Day 2, she developed hypovolemic shock and cardiopulmonary arrest. She was resuscitated and received exchange transfusion but died 57 hours after birth. No autopsy was obtained.

## 3. Discussion

Differential diagnosis of neonatal skin filtrates includes various malignancies [14]. LCH should be considered in the differential diagnosis of widespread cutaneous lesions in neonates. This prematurely born female neonate was diagnosed with disseminated cutaneous LCH lesions. The cutaneous lesions resembled severe burns, with sparse healthy skin areas and the blackened eruptions in the hands and feet were similar to those described previously [11] in a case with purpuric/necrotic papules, which were most prominent on the plantar feet surfaces. Although no pulmonary or bone lesions were apparent on X-rays on her admission, she was in respiratory failure and since the patient lived only for <3 days, no CT scan was carried out to examine the detailed lung lesions. Also, considering her hypoalbuminemia and thrombocytopenia which may indicate the risk organ involvement, it is possible that this neonate could be a case of multisystem disease. Although her LCH presumably developed* in utero*, the results of fetal ultrasonography were normal and did not indicate hydrops fetalis. The likely cause of death was hypovolemic shock as is often seen in patients with extensive burns and pulmonary failure. Unfortunately, because no autopsy was permitted, we could not confirm histopathologically if she actually had multisystemic LCH with involvement of organs other than the skin.

Although cutaneous lesions associated with LCH in infants are often self-healing [5], evidence suggests that the occurrence of these lesions in association with premature birth is not benign [8–12] (Table 1). As summarized, in this case and in six other cases identified in the literature, the majority of premature neonates with congenital cutaneous LCH lesions had a multisystem disease and poor outcome (Table 1). These poor outcomes were mostly associated with pulmonary failure or multiorgan failure, with two cases of hydrops fetalis. Skin lesions were variable, from diffuse cutaneous nodules [11], isolated vesiculopapulomacular rash [12], generalized vesicles [9], papular rash [13], and disseminated burn-like lesions (present case). Of the 2 cases associated with hydrops fetalis, one died early (36 hours), while the other in 12 days [8, 9]. Exceptionally, a premature baby with cutaneous lesions at birth (albeit less extensive than in our case) survived the neonatal period [13]. The prognosis may be affected by the severity or degree of cutaneous lesions at birth, as well as by subsequent multiorgan involvement. In addition, lack of bone involvement might have played a role, because it was recently reported to be a previously unrecognized unfavorable prognostic factor in patients with multisystem, risk organ-positive LCH [15].

Because the life span tends to be very short in fatal cases, it is unknown whether there is sufficient time to consider chemotherapy for LCH. The limited time available for therapy presents challenges for the management of premature babies with congenital LCH. Regarding the biological variables affecting the outcome of LCH, expressions of E-cadherin and CD56 in CD1a^+^ cells may play a role. Absent expression of E-cadherin in LCH is thought to contribute to an aggressive clinical course [6]. CD56 expression has also been reported in cases of Langerhans cell sarcoma [16]. In our patient, E-cadherin expression was not observed, providing further evidence that the absence of E-cadherin is important for the progression of LCH lesions [17]. However, the effects of CD56 expression in CD1a^+^ cells have yet to be confirmed, because CD56 expression was not observed in our patient.

In summary, LCH should be considered in the differential diagnosis of disseminated and hemorrhagic vesicular cutaneous lesions in neonates. To confirm the diagnosis, prompt skin biopsy is required. It should be noted that not all congenital cutaneous lesions associated with LCH are benign and self-healing, and the outcome is affected by multiple factors. Particularly, premature neonates with extensive congenital cutaneous LCH may be fatal, because of associated multisystem organ involvements. In these cases immediate intensive care with LCH-oriented therapeutic measures is necessary to improve the chance of survival.

## Figures and Tables

**Table 1:** Outcomes of premature neonates with congenital cutaneous LCH lesions at birth.

Author (reference number)	Gender	GA (wks)	Clinical features of LCH involvement	Outcome (survival)
Vade et al. [10]	F	35	Skin, hepatosplenomegaly, pulmonary failure	Died (9 days)
Gee et al. [11]	M	33	Skin, pulmonary failure	Died (36 hrs)
Aviner et al. [12]	F	32	Skin, followed by multiorgan failure	Died (26 days)
Lee et al. [8]	M	36	Hydrops fetalis, skin, multiorgan failure	Died (12 days)
Cheng et al. [9]	M	33	Hydrops fetalis, skin, pleural effusion	Died (36 hrs)
Herbrüggen et al. [13]	M	35	Skin, at 3 months, pulmonary and thymic mass	Alive
Present case	F	33	Skin, hypovolemic shock, pulmonary failure	Died (57 hrs)

GA: gestational age, M: male, F: female, wks: weeks, and hrs: hours.
